# Investigating the Effect of Perforations on the Load-Bearing Capacity of Cardboard Packaging

**DOI:** 10.3390/ma17174205

**Published:** 2024-08-25

**Authors:** Kacper Andrzejak, Damian Mrówczyński, Tomasz Gajewski, Tomasz Garbowski

**Affiliations:** 1 Werner Kenel Sp. z o.o., Mórkowska 3, 64-117 Krzycko Wielkie, Poland; kacper.andrzejak@wernerkenkel.com.pl; 2 Doctoral School, Department of Biosystems Engineering, Poznań University of Life Sciences, Wojska Polskiego 28, 60-637 Poznań, Poland; damian.mrowczynski@up.poznan.pl; 3 Institute of Structural Analysis, Poznań University of Technology, Piotrowo 5, 60-965 Poznań, Poland; tomasz.gajewski@put.poznan.pl; 4 Department of Biosystems Engineering, Poznań University of Life Sciences, Wojska Polskiego 50, 60-627 Poznań, Poland

**Keywords:** corrugated cardboard, perforations, box compression test, finite element method, strength analysis

## Abstract

The impact of perforation patterns on the compressive strength of cardboard packaging is a critical concern in the packaging industry, where optimizing material usage without compromising structural integrity is essential. This study aims to investigate how different perforation designs affect the load-bearing capacity of cardboard boxes. Utilizing finite element method (FEM) simulations, we assessed the compressive strength of packaging made of various types of corrugated cardboards, including E, B, C, EB, and BC flutes with different heights. Mechanical testing was conducted to obtain accurate material properties for the simulations. Packaging dimensions were varied to generalize the findings across different sizes. Results showed that perforation patterns significantly influenced the compressive strength, with reductions ranging from 14% to 43%, compared to non-perforated packaging. Notably, perforations on multiple walls resulted in the highest strength reductions. The study concludes that while perforations are necessary for functionality and aesthetics, their design must be carefully considered to minimize negative impacts on structural integrity. These findings provide valuable insights for designing more efficient and sustainable packaging solutions in the industry.

## 1. Introduction

The packaging sector heavily relies on materials such as paper, plastic, glass, wood, and metal, with paper and corrugated cardboard dominating, due to their advantageous properties and cost-effectiveness [1]. Corrugated cardboard is particularly valued for its high strength-to-weight ratio and recyclability, making it an environmentally friendly choice [2]. Its ability to be processed efficiently and formed into various shapes without extensive manual labor gives it a competitive edge in the fast-paced packaging industry [3].

A key feature of corrugated cardboard is its recyclability, which aligns with the concept of a circular economy. Its ability to be recycled makes it an environmentally friendly material, which is particularly significant in the context of global efforts toward sustainable development. Cardboard packaging has a high strength-to-weight ratio and can be transported in a compressed form, allowing for the transport of a larger quantity of packaging, compared to plastic or wooden containers.

Advanced processing machines and the ease of working with corrugated cardboard shorten the time required to complete new projects, giving it a significant advantage over other materials that require special molds or manual labor. The technology for producing corrugated cardboard is continuously evolving, enabling the creation of increasingly sophisticated and complex packaging structures. Cardboard boxes are versatile—they are used both for transporting goods and displaying them on store shelves [4]. The rapidly growing e-commerce industry also appreciates corrugated cardboard, which can be observed during online shopping. The popularity of online shopping has increased the demand for durable yet lightweight packaging that protects products during transport while minimizing shipping costs [5,6].

The popularity of corrugated cardboard packaging brings challenges related to selecting the appropriate raw material and maintaining a balance between aesthetics and cost. Protective packaging for transportation does not always require high durability, and aesthetics are often of lower importance. These packages are usually made of gray cardboard, with prints mainly serving to describe the contents or to identify the product. The production of protective packaging focuses on cost optimization, often meaning the selection of cheaper materials and simpler production processes [4,7]. In contrast, retail packaging, intended for display on store shelves, must be attractive and catch customers’ attention. This is achieved through well-designed graphics and appropriately matched perforations that enable an appealing presentation of the products. Improperly chosen perforations can ruin the packaging aesthetics, negatively impacting the perceived quality of the product.

**Figure 1:**
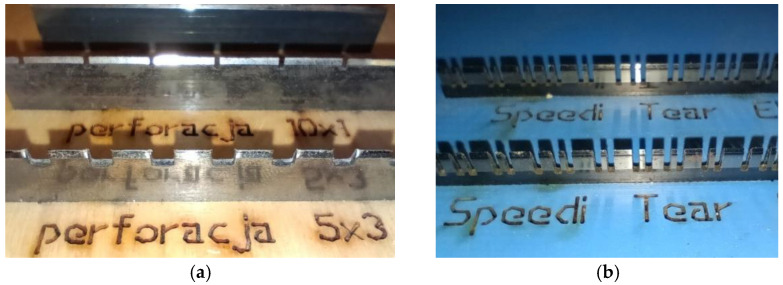
Knives for (**a**) 10 × 1 mm (upper) and 5 × 3 mm (lower) standard perforations and for (**b**) Speedi Tear perforations.

The standard and simultaneously most popular perforations are defined by two numbers, where the first indicates the length of the cut and the second the length of the bridge, or gap, in the cut, e.g., 7/3. Cutting sequences are adjusted based on the wave profile and the quality of the paper. For B flutes, a 7/3 perforation is suitable, while for five-layer BC-profile cardboard, a 15/8 perforation is a better option. Exemplary knives of standard perforations are demonstrated in Figure 1a.

Using raw materials with a high content of cellulose requires longer cuts and/or shorter bridges, as it is less prone to tearing. Choosing the appropriate perforation is crucial because it affects the durability and functionality of the packaging [8]. When the load is carried by the packaging rather than the product, the bridges act as pillars, supporting and stiffening the structure and preventing product damage. A different method is used when designing packaging where the product can carry the load—some strength is sacrificed for better aesthetics by using smaller bridges.

An alternative between the standard perforation and the reverse cut could be the so-called Speedi Tear perforation, which finds a balance between maintaining packaging strength and the quality of tearing. This type of perforation resembles the standard type, but the cutting and bridging sequence is slightly more complex, as shown in Figure 1b.

**Figure 2:**
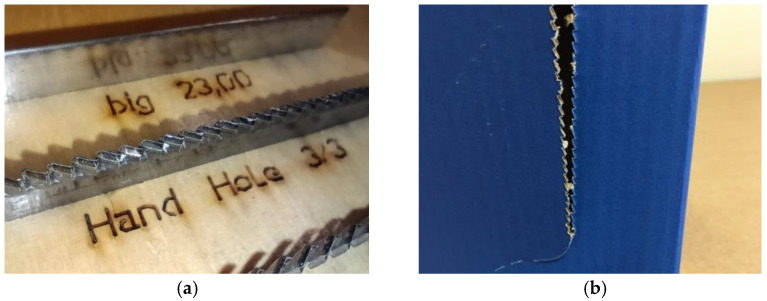
(**a**) Cascade knife and (**b**) cascade perforation on the box.

Other solutions worth mentioning include the cascade perforation, which is characterized by the high ease of tearing with minimal force, although it compromises the finish aesthetics, as shown in Figure 2.

**Figure 3:**
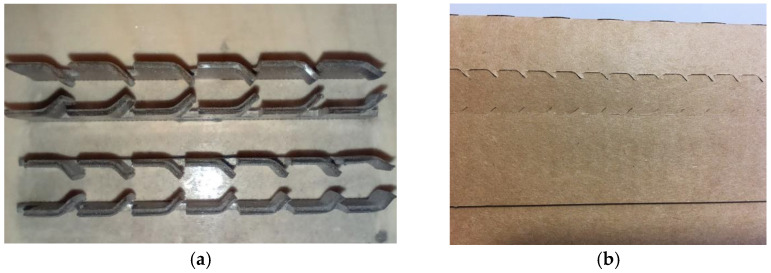
(**a**) Zipper knife and (**b**) zipper perforation.

The zipper perforation, often used in the e-commerce industry, allows for the easy opening of the packaging when combined with tear tape. It is used when quick and simple access to the contents is crucial for the end customer, as shown in Figure 3.

An important factor is understanding how specific patterns of perforations can decrease the final strength of the box. This knowledge is not readily available in scientific literature. Therefore, this paper addresses this topic and demonstrates the mechanical analysis of the most common cases of packaging with perforations.

Existing literature extensively covers the mechanical properties of corrugated cardboard and its behavior under different loading conditions [9,10,11,12,13,14]. Studies by Garbowski et al. [15] and Mrówczyński et al. [16] have provided significant insights into the factors influencing the strength and durability of corrugated cardboard packaging. Garbowski et al. [15] focused on the effects of analog and digital crease lines on mechanical parameters, revealing the critical role of crease design in packaging performance. Mrówczyński et al. [16] extended this understanding by analyzing the strength of cardboard packaging subjected to dynamic transport loads, which highlighted the practical implications of packaging design on its protective capabilities during shipping and handling [5,6,16,17].

However, a notable gap in literature is the lack of detailed studies on the specific impact of perforations on the structural integrity of cardboard packaging. Perforations are commonly used to enhance the functionality and aesthetic appeal of packaging, but their introduction can compromise the material’s load-bearing capacity. Research by Garbowski et al. [8] touched upon the compressive strength of boxes with various perforations, yet comprehensive quantitative data on how different perforation patterns affect packaging strength have still not been fully explored.

This study addresses this gap by employing the finite element method (FEM) to simulate and analyze the compressive strength of corrugated cardboard packaging with various perforation patterns. This popular numerical method was widely used in the precise estimation of various load-bearing capacities of corrugated cardboard packaging [9,10,11,12,18,19]. In the past, analytical methods were also employed to estimate the load-bearing capacity of cardboard packaging [20]. Numerous equations were developed, such as the McKee formula [21], which is still widely used by industry box designers today. However, many improvements have been made to enhance the accuracy of these estimations [22,23,24,25]. These advancements include refining the original equations [25] and incorporating more variables that affect packaging strength [8], leading to more precise and reliable predictions in various practical applications [26,27].

Several studies in scientific literature address the impact of perforations on the load-bearing capacity of cardboard packaging. Important works include Šarčević et al. [28], which explores the validation of methods for testing perforated cardboard. Another study, again by Šarčević et al. [29], examines how perforations affect the bending stiffness of corrugated cardboard. Additionally, a paper by the same authors [30] discusses the reduction in compressive strength due to perforations. These studies contribute valuable insights into understanding and mitigating the effects of perforations on packaging performance. However, they do not exhaust the topic, and therefore, this work aims to fill this gap in the research.

This study includes multiple common types of corrugated cardboard and a range of packaging dimensions to ensure a broad applicability of the findings. Mechanical testing was conducted to obtain accurate material properties, which were then used in the FEM simulations. The results provide a detailed understanding of how perforation patterns influence packaging strength, offering valuable guidelines for optimizing the design to achieve a balance between functionality, aesthetics, and structural integrity. This research contributes to the broader field of packaging technology by enhancing knowledge based on the structural performance of corrugated cardboard, thereby supporting the development of more effective and sustainable packaging solutions.

## 2. Materials and Methods

### 2.1. Workflow of the Study

This research aims to investigate the impact of perforation patterns on the compressive strength of corrugated cardboard packaging. Several perforation patterns that are commonly used in the packaging industry were selected, and the packaging compressive strength was numerically predicted using the finite element method for each shape. The study considered scenarios where perforation was present on a single wall, three walls, or all four side walls of the packaging. All perforation patterns considered are described in Section 2.3. It was assumed that the perforation knife setup remained unchanged (5/5 standard knife). In addition to the perforated packaging, reference cases without perforation were also modeled, and the percentage decrease in compressive strength was determined for each package with a specific perforation pattern.

A numerical approach using the finite element method (FEM) was employed to calculate the package’s compressive strength to achieve comparable results. Material data for the corrugated cardboard were obtained using the BSE system [31], which performs the edge-crush test (ECT) according to ISO 3037:2022 [32], four-point bending in the machine and cross-machine directions (MD and CD) according to ISO 5628:2019 [33], torsion in MD and CD, and the shear stiffness test. In the BSE system, five samples of each cardboard type were tested to determine their material properties. Five samples were used to obtain statistically significant properties, and the average values were used as the representatives in the calculations. This enabled the acquisition of a complete set of material constants for modeling in the FEM environment. 

To generalize the research findings, several types of corrugated cardboard were also considered, namely E, B, C, EB, and BC flutes. (Flute heights are shown in Table 1.) The selected cardboards consisted of both virgin and recycled fibers. For each perforation pattern, an analysis was conducted for all types of corrugated cardboards. Additionally, several different package sizes were selected, i.e., 300×200×200, 300×200×250, 300×200×300, 250×250×250, 350×350×350, and 450×450×450 mm. A total of 240 numerical analyses were performed: 8 packaging designs (1 reference and 7 with perforations) × 5 types of corrugated cardboard × 6 different packaging dimensions.

**Figure 4:**
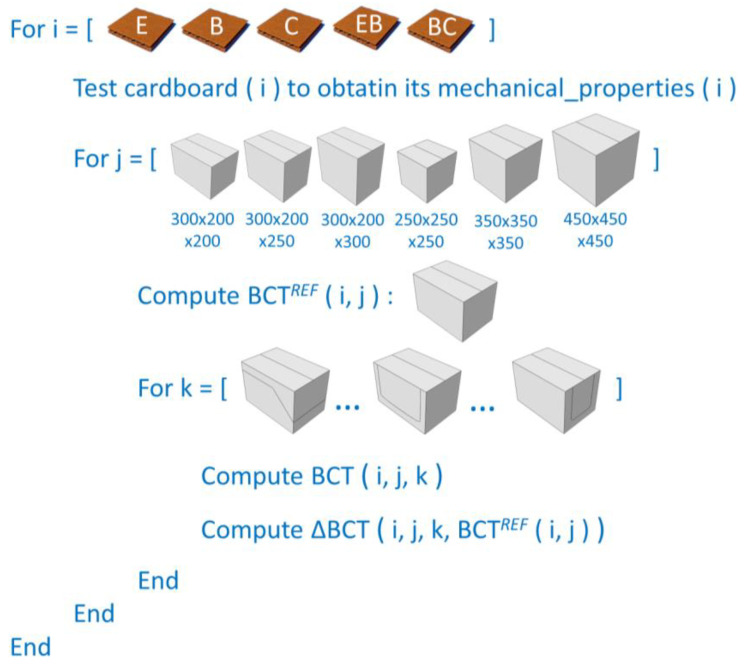
The schematic approach to estimating the decrease in load-bearing capacity of different packaging made of various cardboards through the utilization of diverse perforation configurations.

Figure 4 schematically illustrates the scope of the study. The subscript i represents the selected types of corrugated cardboards; subscript j refers to different packaging dimensions; and subscript k represents packaging with different perforation patterns. In summary, for each possible combination of (i, j, k), the packaging’s compressive strength and its decrease relative to the reference design without perforation (i, j) were calculated.

Detailed information is provided in the subsections: in Section 2.2, the tests for different cardboards necessary to perform accurate FEA calculations are discussed; in Section 2.3, the numerical models used are described. In Section 3 and Section 4, the results with a particular focus on the dependence of compressive strength on the aspect ratio of the box, perforation pattern, and type of corrugated cardboard used are presented and discussed.

### 2.2. Mechanical Properties of Corrugated Cardboards Analyzed

In the presented work, the mechanical testing of corrugated cardboard was an important part of the study. Only reliably obtained input data for material modeling enabled the achievement of accurate calculation results. Similar to the studies conducted in [15,16,34], all types of corrugated cardboards were subjected to the following tests: four-point bending in the machine direction (MD) and the cross-machine direction (CD), edge-crush testing, transverse shear stiffness testing, and torsion testing in both directions, MD and CD. During the mechanical tests, the grammage and thickness of the cardboard were also measured. In separate tests, using a moisture analyzer (Axis, Gdansk, Poland), the moisture contents of the cardboards were determined. Table 1 summarizes all physical properties of the boards used.

**Figure 5:**
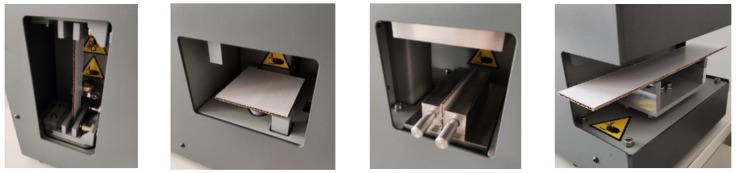
Testing sockets of the measuring device used for determining the mechanical properties of corrugated cardboards (from left: torsion, transverse shearing, ECT, and four-point bending) (source: authors’ own work).

The aforementioned mechanical tests of the corrugated cardboards were conducted using the multifunctional device, namely the BSE system (FEMat, Poznan, Poland) [31], which integrates the essential and necessary mechanical tests to model the cardboard robustly. The testing sockets of the BSE machine used for the tests are shown in Figure 5. Once the samples were placed, all tests were performed simultaneously. Conducting the tests simultaneously not only accelerated the entire process but also ensured that the samples were tested under the exact same moisture and temperature conditions. Here, the cardboard samples were conditioned according to the TAPPI standard [3]; a humidity of 50% and a temperature of 23 °C were maintained in the climate chamber. To obtain statistically significant results for each type of cardboard (wave profile), the tests were carried out on 5 sets of samples. The samples were cut with a hand punch, in addition to the ECT samples, which were cut with a pneumatic device, in order to obtain a high-quality sample edge, which is crucial in ECT.

The constitutive model of corrugated cardboard used was assumed to be orthotropic elastic and ideally plastic, with an addition of Hill’s plasticity potential in the machine material direction to differentiate plastic behaviors between MD and CD [35]. The approach is state-of-the-art in modeling corrugated structures, enabling practical efficiency and computational accuracy, as shown in [15,16,34] and in many others. A systematic testing campaign enabled us to obtain the average material constants for all cardboards used for the assumed models of boxes without and with perforations, as shown in Table 2. The first columns contained the elastic parameters: the Young’s moduli in MD and CD, $E_{1}$ and $E_{2}$, respectively, Poisson’s ratio $\nu_{12}$, Kirchhoff’s modulus $G_{12}$, and transversal shear stiffnesses $G_{13}$ and $G_{23}$. The last two columns contained the plastic parameters: the yield strength $\sigma_{0}$ and $R_{11}$, which is the yield strength factor applied in the machine direction, according to the Hill’s potential [35].

### 2.3. Finite Element Models of Corrugated Cardboard Boxes with Perforations

**Figure 6:**
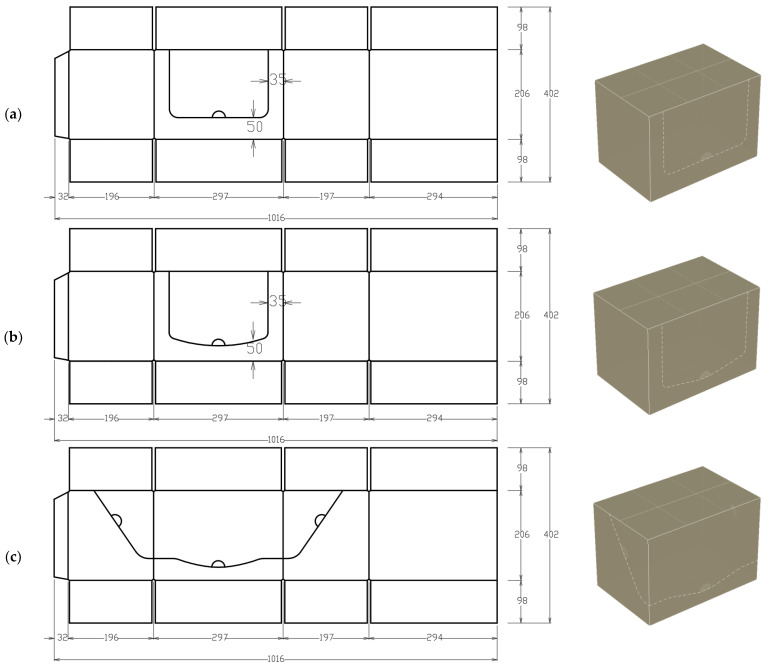

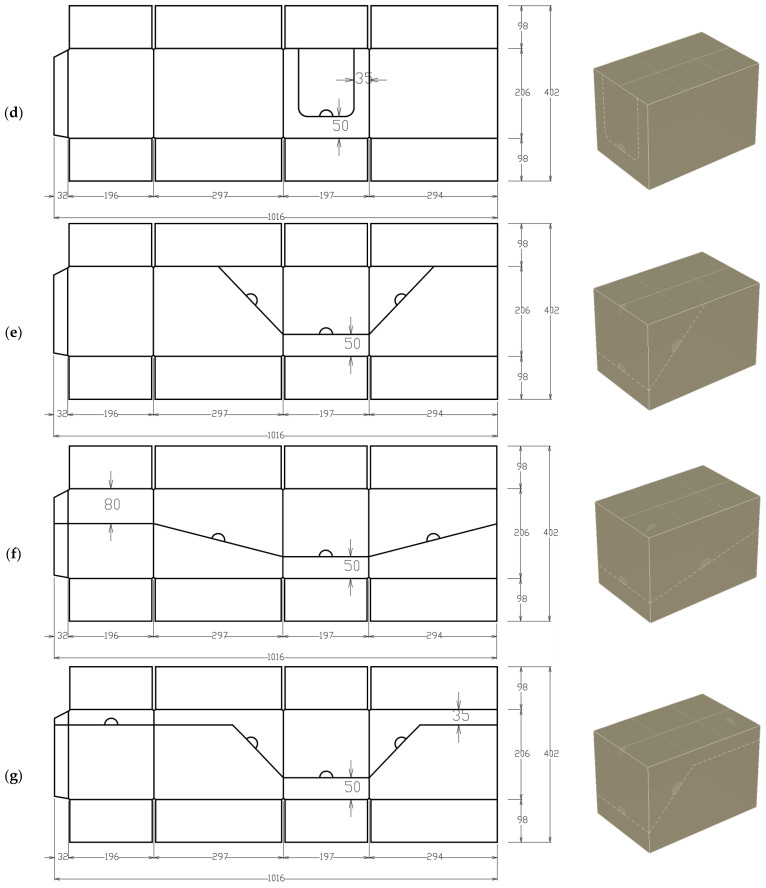
Grids of the packaging with perforations utilized in the study, along with their three-dimensional assemblies: (**a**) C1, (**b**) C2, (**c**) C3, (**d**) C4, (**e**) C5, (**f**) C6, and (**g**) C7.

Box compression tests of corrugated cardboard packaging were simulated in commercial software, (10.4, FEMat, Poznan, Poland) based on the finite element method [31]. The values from Table 2, which were obtained by performing mechanical tests on cardboard using a BSE machine, were used as material data, as shown in Figure 5. The analyses were performed for 6 box dimensions, 5 types of cardboards, and 8 cases of perforations (1 reference case without perforation and 7 different types of perforations), which ultimately resulted in 240 unique numerical models of packaging. In Figure 6, 2D and 3D views of the 300×200×200 mm packaging with the considered perforation patterns, marked with symbols C1–C7, are presented. The selected perforations are the most common perforations used in the packaging industry. Three perforation patterns out of seven occurred on only one wall (C1, C2, and C4), two patterns occurred on three walls (C3 and C5), and the remaining two occurred on all four walls of the box (C6 and C7). The perforation patterns differed in shape, rounding, and position on the height of the packaging.

During the simulation of the box compression test, the bottom and top of the packaging were omitted because the load was carried by the sidewalls. However, the bottom and top flaps were taken into account by adopting appropriate boundary conditions, i.e., blocking out-of-plane edge displacements of the sidewalls. The load-bearing capacity was calculated, taking into account the buckling of the walls. In all analyses, the six-node triangular shell elements were used. Due to different packaging dimensions and perforation types, the number of elements changed. For instance, for a 300×200×200 mm box with C4 perforation, the model consisted of 2377 nodes and 1130 triangular elements. In the FEM analyses, the kinetic displacement was applied at the top edges of the box, and the maximal reaction force was sought, which represents the box compression strength.

## 3. Results

The load-bearing capacity of perforated packaging depends on many factors, not just the patterns of the perforations. Other important factors include the dimensions of the packaging and the corrugated cardboard used. Therefore, in this scientific study, in addition to varying the perforation patterns, the dimensions of the packaging and the type of corrugated cardboard were also varied.

In the first part, shown in Table 3, the results for packaging with a fixed base of 300×200 mm and varying heights of 200, 250, and 300 mm were presented. The table shows the compressive strength values obtained using FEM for the reference packaging and perforated packaging, as well as the normalized values (ratio of the perforated packaging to the reference packaging).

In the second part, shown in Table 4, the results for cubic packaging with variable side lengths made from the same corrugated cardboard (B-profile wave) are presented. This includes cubic packaging, with side lengths of 250, 350, and 450 mm. As before, the compressive strengths of the packaging and the normalized values relative to the reference packaging are shown.

In the third part, shown in Table 5, for packaging with the dimensions 300×200×200 mm, the compositions of the corrugated cardboard were varied, utilizing E, B, C, EB, and BC flutes. As before, the compressive strengths of the packaging and the normalized values relative to the reference packaging are shown.

## 4. Discussion

In the paper, the results obtained from experimental work and numerical calculations enabled an assessment of the impact of perforation patterns on the load-bearing capacity of corrugated cardboard packaging.

Firstly, rectangular packaging made from B-profile wave corrugated cardboard with a base ratio of 3:2 and varying heights from 200 to 300 mm were analyzed, as shown in Table 3. No trend was observed in the results with increasing packaging height. For packaging with dominant perforations on the longer side (C1 to C3 cases), no trend was found, regardless of different perforation designs. Load-bearing capacity reductions compared to the reference packaging (without perforations) oscillated around 26 ± 2.5%. For packaging with dominant perforations on the shorter side (C4) and perforations extending to perpendicular longer walls (C5–C7), an increasing trend in load-bearing capacity reduction was observed. The reductions ranged from approximately 14.5% for packaging with perforations on the shorter side to 41% for packaging with perforations on three sides, including bi-line perforation patterns on the longitudinal walls.

Next, cubic packaging with heights ranging from 250 mm to 450 mm were examined, as shown in Table 4. For packaging C1 to C3, slight increases in load-bearing capacity reductions were noted, ranging from approximately 20% to 28%. Moreover, within the C1–C3 group, the load-bearing capacity reduction decreased by a few percentage points with increasing packaging size. For example, for the C1-250 mm packaging, the reduction was 22.5%; for C1-350 mm, it was 20.5%; and for C1-450 mm, the decrease was 18.8%. Similar trends were observed for C4–C7, where load-bearing capacity reductions increased in subsequent columns (averaging around 18%, 22%, 29%, and 44%) and decreased in subsequent rows. For example, for the C7 packaging with bi-line perforation patterns on the perpendicular longer wall, the reductions were 45% for C7-250 mm, 43.5% for C7-350 mm, and 42.8% for C7-450 mm.

In the next part of the study, the impacts of different types of cardboards on the load-bearing capacity of a selected rectangular packaging were verified, as shown in Table 5. It was found that using thicker cardboards only slightly affected the reduction in load-bearing capacity, compared to the reference packaging (without perforations). This finding applied to all analyzed perforation patterns from C1 to C7. The average values of load-bearing capacity reductions, calculated by columns, were approximately 27%, 24%, and 28% for C1, C2, and C3 and 15%, 19%, 33%, and 37% for C4–C7, with variations depending on the type of cardboard used amounting to 2–3 percentage points. In Table 5, for C1–C3, there was no horizontal correlation, similar to the results in Table 3. For C4–C7, horizontal correlation was observed, showing increases in reductions in subsequent columns, with average values slightly lower than those in Table 4 (few percentage points).

In summary, the reductions in the load-bearing capacity of perforated packaging, compared to corresponding packaging without perforations, ranged from 14% to 43%, depending on the perforation design. The smallest reduction occurred when the perforation appeared on only one wall (C1, C2, and C4 cases). The worst cases were when the perforations extended along all four walls and crossed the vertical edge of the box (C6 and C7 cases). The type of cardboard used did not significantly influence the percentage reduction in the load-bearing capacity, nor did its thickness or the number of cardboard walls (single or double-wall).

## 5. Conclusions

In this study, the impact of different perforation patterns on the load-bearing capacity reduction of packaging, compared to the same packaging without perforations, was investigated. Common perforation designs were selected for analysis. Material tests were conducted on five types of cardboards with different wave profiles; the tests included 4-point bending and edge-crush tests, as well as less conventional tests, such as torsion and shearing, to obtain material properties for constitutive modeling. Subsequently, packaging load-bearing simulations were performed, representing column compression tests. Finite element method computations utilized an orthotropic material law with plasticity to model corrugated cardboards.

The results presented in the article demonstrated that the perforation pattern significantly influences the percentage reduction in the load-bearing capacity of the packaging. Also, it was shown that the percentage reduction in the load-bearing capacity is minimally dependent on the dimensions of the packaging. Additionally, the results suggested that the percentage reduction in the load-bearing capacity is not influenced by the type of cardboard used for the packaging. The reductions in the load-bearing capacity of the perforated packaging considered ranged from 14% to 43%, depending on the perforation design. The smallest reduction occurred when the perforation appeared on only one wall, while the worst cases were when the perforations extended along all four walls and crossed the vertical edge of the box.

These findings demonstrate the critical importance of perforation pattern in determining the structural integrity of corrugated cardboard packaging and may extend the knowledge of cardboard box designers. The practical value of the results achieved is considerable, as this type of knowledge is currently lacking in scientific literature and is in high demand among designers of corrugated cardboard packaging. The results provide valuable insights for optimizing packaging designs to mitigate load-bearing capacity reductions while considering different material properties and perforation configurations. 

## Figures and Tables

**Table 1:** Physical properties of corrugated cardboards used in the study.

Grade	Grammage (g)	Thickness (mm)	Moisture (%)
E	349	1.58	7.14
B	410	3.00	6.77
C	449	4.10	6.94
EB	789	4.47	7.08
BC	673	6.76	6.92

**Table 2:** Material constants used in the constitutive models of cardboards used in the study.

Board	$E_{1}$	$E_{2}$	$\nu_{12}$	$G_{12}$	$G_{13}$	$G_{23}$	$\sigma_{0}$	$R_{11}$
	(MPa)	(MPa)	(–)	(MPa)	(MPa)	(MPa)	(MPa)	(–)
**E**	3010	1192	0.472	1762	3.0	2.7	2.57	0.95
**B**	1494	713	0.430	777	3.5	5.0	1.49	0.75
**C**	1057	641	0.381	689	2.1	5.5	1.15	0.59
**EB**	1367	753	0.400	732	6.5	9.7	2.29	0.65
**BC**	562	449	0.332	402	3.2	7.9	1.19	0.50

**Table 3:** Finite element estimations of the compression strengths of boxes made of B flute cardboard, with varying heights and with different perforation patterns.

Dimensions (mm)	B Flute	Reference	C1	C2	C3	C4	C5	C6	C7
300×200×200	BCT (kN)	1284	953	983	932	1106	1057	871	802
	Δ BCT (%)	–	−25.8	−23.4	−27.4	−13.9	−17.7	−32.2	−37.5
300×200×250	BCT (kN)	1326	1014	1008	980	1119	1113	964	824
	Δ BCT (%)	–	−23.5	−24.0	−26.1	−15.6	−16.1	−27.3	−37.9
300×200×300	BCT (kN)	1361	998	993	973	1156	1116	1022	803
	Δ BCT (%)	–	−26.7	−27.0	−28.5	−15.1	−18.0	−24.9	−41.0

**Table 4:** Finite element estimations of the compression strengths of cuboid boxes made of B flute cardboard, with varying sizes and with different perforation patterns.

Dimensions (mm)	B Flute	Reference	C1	C2	C3	C4	C5	C6	C7
250×250×250	BCT (kN)	1477	1145	1137	1070	1191	1103	1024	813
	Δ BCT (%)	–	−22.5	−23.0	−27.6	−19.4	−25.3	−30.7	−45.0
350×350×350	BCT (kN)	1620	1288	1285	1221	1336	1275	1164	915
	Δ BCT (%)	–	−20.5	−20.7	−24.6	−17.5	−21.3	−28.1	−43.5
450×450×450	BCT (kN)	1709	1387	1383	1307	1430	1367	1247	978
	Δ BCT (%)	–	−18.8	−19.1	−23.5	−16.3	−20.0	−27.0	−42.8

**Table 5:** Finite element estimations of the compression strengths of a 300×200×200 box made of different cardboards and with different perforation patterns.

Profile Wave		Reference	C1	C2	C3	C4	C5	C6	C7
E	BCT (kN)	746	552	567	536	633	612	511	451
	Δ BCT (%)	–	−26.0	−24.0	−28.2	−15.1	−18.0	−31.5	−39.5
B	BCT (kN)	1284	953	983	932	1106	1057	871	802
	Δ BCT (%)	–	−25.8	−23.4	−27.4	−13.9	−17.7	−32.2	−37.5
C	BCT (kN)	1676	1202	1243	1192	1408	1336	1101	1048
	Δ BCT (%)	–	−28.3	−25.8	−28.9	−16.0	−20.3	−34.3	−37.5
EB	BCT (kN)	2888	2135	2208	2098	2488	2372	1942	1819
	Δ BCT (%)	–	−26.1	−23.5	−27.4	−13.9	−17.9	−32.8	−37.0
BC	BCT (kN)	3052	2199	2281	2264	2611	2482	2061	2006
	Δ BCT (%)	–	−27.9	−25.3	−25.8	−14.4	−18.7	−32.5	−34.3

## Data Availability

The data presented in this study are available upon request from the corresponding author.
